# Stakeholders’ perceptions regarding a salt reduction strategy for India: Findings from qualitative research

**DOI:** 10.1371/journal.pone.0201707

**Published:** 2018-08-06

**Authors:** Priti Gupta, Sailesh Mohan, Claire Johnson, Vandana Garg, Sudhir Raj Thout, Roopa Shivashankar, Anand Krishnan, Bruce Neal, Dorairaj Prabhakaran

**Affiliations:** 1 Centre for Chronic Disease Control, New Delhi, India; 2 Public Health Foundation of India, Gurgaon, Haryana, India; 3 The University of Sydney, Sydney, Australia; 4 The George Institute for Global Health, Sydney, Australia; 5 George Institute for Global Health, Hyderabad, India; 6 All India Institute of Medical Sciences, New Delhi, India; 7 Royal Prince Alfred Hospital, Sydney, Australia; 8 Imperial College, London, United Kingdom; ESIC Medical College & PGIMSR, INDIA

## Abstract

**Background:**

Scientific evidence indicates that high dietary salt intake has detrimental effects on blood pressure and associated cardiovascular disease (CVD). However, limited information is available on how to implement salt reduction in low and middle-income countries (LMICs) such as India, where the burden of hypertension and CVD is increasing rapidly. As part of a large study to create the evidence base required to develop a salt reduction strategy for India, we assessed the perspectives of various stakeholders regarding developing an India specific salt reduction strategy.

**Methods:**

A qualitative research design was deployed to elicit various stakeholder’s (government and policy-related stakeholders, industry, civil Society, consumers) perspectives on a salt reduction strategy for India, using in-depth interviews (IDIs) and focus group discussions (FGDs). We used an inductive approach for data analysis. Data were analyzed using thematic content analysis method.

**Results:**

Forty-two IDIs and eight FGDs were conducted with various stakeholders of interest and relevance. Analysis indicated three major themes: 1. Barriers for salt reduction 2. Facilitators for salt reduction; 3. Strategies for salt reduction. Most of the stakeholders were in alignment with the need for a salt reduction programme in India to prevent and control hypertension and related CVD. Major barriers indicated by the stakeholders for salt reduction in India were social and cultural beliefs, a large unorganized food retail sector, and the lack of proper implementation of even existing food policies. Stakeholders from the food industry reported that there might be decreased sales due to salt reduction. Major facilitators included the fact that: salt reduction is currently a part of the National Multi-Sectoral Action Plan for the prevention and control of NCDs, salt reduction and salt iodine programme are compatible, and that few of the multinational food companies have already started working in the direction of initiating efforts for salt reduction. Based on the barriers and facilitators, few of the recommendations are to generate awareness among consumers, promote salt reduction by processed food industry, and implement consumer friendly food labelling.

**Conclusions:**

In this study of multiple key influential stakeholders in India, most of the stakeholders were in alignment with the need for a salt reduction programme in India to prevent and control hypertension and related CVD. The development and adoption of the National Multi-sectoral Action Plan to reduce premature non-communicable diseases (NCDs) in India, provides a potential platform that can be leveraged to drive, implement and monitor salt reduction efforts.

## Introduction

Evidence from many studies indicates that high dietary salt intake increases blood pressure and the risk of hypertension. Excess salt consumption is attributable to 1.65 million deaths worldwide annually [1,2]. The World Health Organization (WHO) has identified a 30% reduction in population salt intake as one of the major targets in its global action plan for the prevention and control of non-communicable diseases and recommends a maximum dietary salt intake of < 5 g/day for adults[3,4]. It is estimated that even a small downward shift of blood pressure at the population level would lead to a larger drop in blood pressure related cardiovascular diseases [5].

In India, hypertension is highly prevalent and responsible for at least 10% of the deaths and is projected to increase rapidly in the future [6,7]. In 2016, Disability Adjusted Life Years (DALYs) lost due to dietary risk factors (including high sodium intake) was 8.9% [8] and there was a 30% increase in DALYs lost due to dietary risks in the last decade [9]. Though the scientific evidence for salt reduction is strong, the data required to translate scientific evidence into public health policy and consequently reduced population salt intake are limited, particularly in low and middle-income countries (LMICs) such as India[10,11].

With the aim of addressing this important gap, we conducted a large multi-component study, to assess salt intake and its sources in India, as well as determine the perspectives of various stakeholders, towards developing the evidence base required to develop a salt reduction strategy for India[12]. The results of the quantitative assessment indicated a salt intake of 9.45 g/day (95% CI 8.45–10.46) and 10.41 g/day (95% CI 9.97–10.84), in Delhi and Andhra Pradesh respectively [13]. This is clearly above the WHO recommended level[4]. The quantitative assessment also indicated that a large amount of salt intake is from home-based foods, which is in contrast to developed countries where most salt comes from processed foods. Thus, there is need to develop salt reduction strategies that are well suited to Indian settings. An expert group constituted by the Food Safety and Standards Authority of India (FSSAI) has agreed with the existing WHO and Indian Council of Medical Research (ICMR) recommendations and called for improved food labelling, gradual reduction in commercial and home-based foods, advertisement ban for foods high in fat, sugar and salt, as well as advocated food reformulation of processed foods [14]. This paper presents the results of the qualitative research component of the study which aimed to assess the perspectives of various stakeholders regarding developing an India specific salt reduction strategy.

## Materials and methods

A qualitative research design was deployed to elicit stakeholder’s perspectives on a salt reduction strategy for India, using in-depth interviews (IDIs) (S1 Appendix) and focus group discussions (FGDs) (S2 Appendix)[15] Stakeholder analysis is a widely recommended tool for gathering information on stakeholders understanding and interest for addressing health policy issues [16,17].

### Participants

The participants comprised key individuals who were working with influential health policy making bodies such as; the central and state governments as well as in the health departments (n = 4); the India Office and the South East Asia Regional Office of the World Health Organization (WHO) (n = 5); the Indian Council of Medical Research (ICMR) (n = 2); the World Bank (n = 1); food manufacturers (n = 6) (processed food manufactures and street vendors); the academia (n = 10); the National Institute of Nutrition (NIN) (n = 3) and the civil society (n = 4). Additionally, community members (n = 7) from the population survey sites in the urban, urban slum and rural areas were also invited to participate[12].

### Sampling

Study participants for the IDIs and FGDs were purposively selected through existing contacts and networks in an effort to secure representation from all stakeholder groups of interest and relevance. Participants selected included government and policy-related stakeholders with a stake in non-communicable disease prevention and control, food industry (multinational companies and street vendors), civil society representatives and consumers. Where the required contacts did not currently exist, they were identified from desk research (through a global search of relevant institutional /organizational websites), and an invitation was made by a senior member of the research team. In every case, participants were contacted directly by telephone or email to seek their participation. They were provided with a participant information sheet, had any of their questions answered and were asked to sign an informed consent form.

### Data collection

Data were collected using semi-structured in-depth interview schedule and by focus group guide. Two research team members facilitated interviews and focus groups, probing participants when issues of particular interest were raised. Prompts were used when issues of interest did not spontaneously arise. All IDIs (except one via phone) were conducted face-to-face and in the respective participant’s workplace.

In-depth interview questions differed slightly between stakeholder groups but largely addressed the following domains of interest: interventions to change consumer behavior, industry actions to reduce salt in their products, and government’s role (i.e., through trade, commerce, marketing, food controls, licensing) in reducing population salt intake.

Eight focus group discussions were conducted among consumers, 3 in South India and 5 in North India. In South India, one FGD each was conducted among the rural, urban, and urban slum populations respectively. In North India, three FGDs were conducted among the rural population while one each was done among urban and urban slum populations respectively. Each FGD comprised of eight participants, of both genders and different age groups. All participants actively participated in the discussions. The three main domains addressed during the FGDs were: consumer perceptions about high salt food, actions to reduce salt in their food and feasibility issues related to reducing salt in the food. These domains for IDIs and FGDs were identified based on the literature review and expert opinion. Prior to data collection, the interview guide containing the domains was pilot tested among five individuals who were not part of the study. In addition, the interview guide was further evolved and refined during the study based on the responses from the previous participants.

### Data preparation and management

IDIs and FGDs were audio recorded, transcribed, translated (if in local language), de-identified and verified. Focal group discussions, and IDIs with consumers were conducted in local languages (Hindi and Telugu). The first author did the Hindi language translation. Telugu language translation was done by other research staff which was back translated and crossed checked by the fifth author. Subsequently, the transcripts were obtained and analyzed. Transcript data were managed using word processing software. Transcripts were labeled to identify participant quotes according to stakeholder groups.

### Data analysis

We employed an inductive approach and used thematic content analysis method to evaluate the transcripts. The first round of analysis involved the first author (PG) reviewing the transcripts verbatim and inductively assigning codes to emergent concepts. By constant comparison, codes were refined until a hierarchy of conceptual codes, and sub-codes emerged. This coding framework was finalized once no new concepts emerged. The second round of analysis involved the fourth author (VG) using the same coding framework to apply codes independently to the transcripts. The research team discussed the coding discrepancies among the two authors and resolved by consensus to optimize inter-coder reliability. All data analysis was done manually.

Codes of similar meaning were clustered to form themes and compared to the transcribed text in an iterative manner, improving the accuracy of data synthesis and interpretation of the meaning. Linkages between themes were also developed at this stage.

This study was approved by the Human Research Ethics Committees of the Centre for Chronic Disease Control in New Delhi and the University of Sydney in Australia as well as by the Indian Health Ministry’s Screening Committee.

## Results

Table 1 has details of the stakeholders interviewed for the study. Out of 57 stakeholders invited for the IDIs, 42 stakeholders participated (73.7%) while of the 72 people selected for FGDs, 60 people participated (83.3%) in the study. From the data analysis of the three main influential stakeholders groups, three major themes were determined: 1. Barriers for salt reduction 2. Facilitators for salt reduction; 3. Strategies for salt reduction. Most of the stakeholders were in alignment with the need for a salt reduction programme in India to prevent and control hypertension and related CVD.

**Table 1:** Participant profile for the IDIs and FGDs.

No	Stake-holder groups	No of people invited to participate in study	No of people interviewed	Mode of data collection
1	Government and policy related stake-holders:			
	• State and central government officials	10	4	In-depth interview
	• International NGO (WHO and World Bank representatives)	8	6	In-depth interview
	• Government research bodies (ICMR, NIN)	7	5	In-depth interview
	• Academia	11	10	In-depth interview
2	Industry			
	• Multi-national companies (MNCs)	6	4	In-depth interview
	• Street vendors	2	2	In-depth interview
3	Civil society (Consumer representatives, *Sarpanches*-local government representatives, local practitioners, teachers and Accredited Social Health Activists (ASHAs)	13	11	In-depth interview
4	Consumers	72	60	Focus group discussion

### Barriers for salt reduction in India

#### 1. Barriers related to cultural and social beliefs

Most participants among the consumers, government representatives and physicians opined that India is a tropical country, where there is excessive sweating and thus a need for a higher salt consumption compared to other countries.

*“Myth that like India is a warm country and as we sweat more*, *we need more salt*. *These kind of myths are there not only in lay people*, *but these are there even in the profession.” (Participant from academia)*

According to industry representatives and nutritionists, salt reduction requires behavioral change which is quite a complex and time-consuming process. Illiteracy in the population and cultural diversity across different states in terms of the type of foods and preparation methods, vary widely within a state, as well as across states. This was identified as the major barrier for creating behavioral change and increasing awareness among consumers.

*“Traditional practices*, *illiterate people*. *In India*, *there is a large section of the population which is not literate*, *they don’t have an attitude to obey*, *and they get a great kick to disobey.” (Participant from academia)*

*“It is important to reduce salt…but kids don’t eat foods with less salt*, *and even some elderly people throw away food if salt is less.” (Participant consumer)*

#### 2. Barriers related to the unorganized food retail sector

All stakeholders felt that the large unorganized food retail sector, i.e., specifically street vendors who are not under the purview of any food regulations, to be a big challenge. They felt that enhancing consumer awareness to exercise healthier choices could be the solution to this problem.

“*Major concern is you have a huge unorganized sector which doesn’t fall under the purview of any regulation.” (Participant from academia)*

*“It’s difficult… as restaurants have fixed menu*. *They have their chefs for presenting it*. *There is a certain pattern*. *So feel it’s particularly the part of the education*, *I don’t think more can be done.” (Participant from the government)*

*“There is no law by which we can interfere with the food content*, *what is served.” (Participant from civil society)*

#### 3. Lack of proper implementation of existing food and dietary policies

Although there are certain food policies in India, they are not properly implemented. Many participants identified that introducing a new food policy in such a milieu where most existing policies aren’t effectively implemented will be less effective and a time-consuming process.

*“In spite of our strict legislation*, *it won’t work out*, *in India*. *The implementation of that law will not happen because of lack of human resources and enforcement.” (Participant from the government)*

*“Policy or law development will take minimum 5–10 years*. *So*, *for immediate action*, *it should be voluntary*. *But*, *we should start meeting people to make law.” (Participant from the government)*

#### 4. The decrease in sales

Most of the stakeholders from the food industry, especially from the unorganized food retail sector had concerns regarding likely decreases in sales as they felt that reduced salt content in the food will affect the consumers’ taste of the food and subsequently have an impact on their sales.

*“People will not eat if there are fewer spices and salt in the street food*.*” (FGD participant)*

*“Industry has an excuse by saying that people like it only with salt*, *people have learned to eat with salt as the industry has provided that*.*” (Participant from civil society)*

### Facilitators for salt reduction in India

#### 1. Salt reduction is a major target in the global and national action plan for reducing non-communicable diseases (NCDs)

Salt reduction is a major target under the recently initiated national multi-sectoral action plan for the prevention and control of non-communicable diseases in India.

*“The Government is now bound to do that because*, *under the NCD global targets*, *salt reduction is one of the key targets and a reporting indicator*. *In the world assembly*, *the health minister will have to say how much has been achieved.” (Participant from research bodies)*

#### 2. Salt reduction and salt iodization are compatible

Stakeholders involved with the iodine fortification programme agreed that if salt intake is reduced, the level of iodine in salt can be increased to meet the objectives of the universal salt iodization programme and that both salt reduction and salt iodization can be cost-effective and compatible.

*“I think this controversy does not exist in the community as much as it exists among so-called scientists*. *I think it’s completely false that we have propagated to eat more salt message*, *the message was always: “eat iodized salt”*, *that message that will still be there eat iodized salt*. *So the message of iodine deficiency programme is not going to change.” (Participant from academia)*

*“Technologically it is feasible to do it*, *it’s not the major issue*, *you are adding 15ppm*, *and you can very well add 7.5 ppm and 10 ppm*. *Switzerland used to do it*, *staggered every five years*, *they used to revise it.”(Participant from academia)*

#### 3. Some food industries are already working on salt reduction

It emerged from the interactions that the food industry, particularly the organized sector is aware of the health impact of excess salt intake and a few of the multinational food companies are already working to reformulate foods to reduce salt content.

*“From next year we will label the sodium on the pack*, *seasoning can be done by microlite salt technology to decrease the sodium*, *but this can be done on dry products only like chips and namkeen*. *(a small savory snack or dish).” (Participant from food industry)*

*“When you look at the food processing industry you have the organized sector*, *you have Kurkure (this is a brand of corn puffs)*, *you have Lay’s chips*, *and things like that*. *Companies like this*, *I am not sure*, *but I am hopeful that they would have some strategy to reduce the salt content in their food because they know that high sodium is a public health concern.” (Participant from food industry)*

#### 4. Increased awareness about salt reduction among the patient population

Findings from the consumers FGDs suggest that awareness regarding the excess salt intake and its harmful health effects was largely known to the hypertensive patients and their families. This can also act as a potential facilitator if appropriately leveraged for improving consumer education.

*“I know that salt should be reduced in our diet*. *Doctor informed about the importance of salt reduction in hypertension*. *Usually I and my family have food with less salt only*.*” (A hypertensive patient)*

*“When I was diagnosed with hypertension*, *doctors informed me to decrease the food with high salt concentration like chips*, *pickles*, *and papad (thin*, *crisp*, *disc-shaped food from the Indian subcontinent).” (A hypertensive patient)*

### Potential strategies for salt reduction in India

#### 1. Generate awareness among the consumers, industry and health professionals

All stakeholders agreed that consumer education is the most important component for a salt reduction effort in India. They indicated that awareness should be generated among all age groups, especially among children and women. In most of the FGDs and consumer interviews, we found that those who have hypertension were aware of salt and its ill effects, but they were unaware about how much salt they should actually consume, how much they are consuming now and how to decrease it. Consumers felt that specific strategies were required to increase awareness, especially on how to reduce the personal salt intake.

*“Mass media is an effective way; internet and social networking websites can help in a move towards consuming low salt… then it will become a fashion to ask for a low salt food item*. *Only then people will.” (Participant from academia)*

*“You know they really listen to the doctors and implement their advice*. *Behaviour change comes very fast if doctors tell them… but many times I have observed that… I don’t know… doctors don’t give that much importance to nutrition.” (Participant from food industry)*

Most of the physicians and nutritionists suggested that salt reduction awareness should be incorporated into overall dietary guidelines and be integrated into general healthy diet plans and not as a standalone dietary strategy.

*“A single component campaign will go into the trash*. *I think it should be the part of a larger campaign*. *Because otherwise*, *people will be sick and tired of… see today its salt*, *tomorrow its sugar and day after it will be something else*, *so it should be embedded in a larger campaign of healthy eating*. *You know it’s simple… salt is bad for blood pressure; something else is bad for something else*, *so you know people tend to get confused*. *People should know what a prudent diet is*. *This is sort of food that is good or bad so they should be taken in certain fashion.” (Participant from academia)*

While the food processing industry seems to be aware of high salt consumption and its adverse health consequences, local street vendors are unaware and need to be educated about salt and its ill effects. For increasing awareness among local street vendors and restaurants, professional bodies of Indian restaurant and street food associations can be involved in this initiative.

*“Create awareness among manufacturers of the processed food or semi-processed foods or restaurants or hotel or street food vendors*.*” (Participant from the government)*

#### 2. Voluntary and gradual reduction of salt by industry

Stakeholders from the food industry, government, academia and the civil society agreed that food industry should be made collaborative partners for initiating salt reduction in India. They opined that making a mandatory law for manufacturing low sodium products will take time and will be difficult to implement.

According to physicians and nutritionists, voluntary and gradual reduction of salt in food products will not affect the cost, taste and the sale of the products and may be a feasible way forward.

*“Very often salt is added for the taste and attraction of the product*, *and they would also be reluctant to reduce the salt because it will squeeze out the taste*, *but a very very gradual and protracted but stage wise reduction would be something that can be done*. *This will make the industry friendlier to the health of its consumer*.*” (Participant from academia)*

*“That is there strong requirement to involve industries; they can actually suggest a lot of things*. *Because a lot of success of the iodization programme*, *I will personally like to attribute to the industry as they have picked it up*. *So*, *you will definitely have to target the most market share industries and see how it could be*. *Once the market leaders are convinced to launch a low sodium product*, *they will attract profit to it.” (Participant from civil society)*

#### 3. Improving nutrition labeling and product reformulation

According to nutritionists, physicians, and academia, the nutrition labeling technique in India is not comprehensive and user-friendly. Given the increased processed food consumption, they felt that having a user-friendly front of pack labeling for all nutritional components will be an effective strategy for enabling healthier food choices. They opined that the policy-makers and the Food Safety and Standards Authority of India (FSSAI) should enforce labeling, and should monitor the enforcement periodically. They were of the opinion that the food industry should bring in newer technologies for reformulation and produce low salt food products.

*“If you look at the labeling laws*, *and labeling on the packs*, *they are really ancient*, *first of all consumers don’t look at the label*, *even if they look at label they don’t understand what to look at and what not to look at*, *third part is they don’t even know what is good and what is bad*. *So*, *labeling should be such that it helps people to make a healthy choice*. *In some countries have very clear nutrition labeling*. *You have choices as they have in the Netherlands*, *which is a tick mark which says it’s a healthy product.” (Participant from the food industry)*

#### 4. Incentives for the food industry to produce low salt products

Many food industry representatives felt that they should be given incentives like tax exemptions on low salt food or provided logos which can be a sign of healthy food. They opined that the Government could make available only low salt food in government hospitals, offices, and other institutions as this will provide a big market to the industry to sustain sales and profits in initial days and then subsequently create awareness among consumers across the society.

*“You have to start from somewhere like in all government canteens this can be used*, *in all government hospitals this low salt food will be used*, *you have to start from somewhere*. *You can make it mandatory in places like defense forces to see the immediate benefits*. *So when critical mass is covered*, *industries will be functional then they will start educating the people also*, *and change will come*. *The Average person will join in the last; he will see that they are not making a fool of me*, *once he is sure he will join*. *As the average person is the most vulnerable section of the society.” (Participant from the food industry)*

*“If you give options to the industry I think that is the best way*, *also give something to them to latch on to… and if you educate consumer and there is demand from consumers comes up the industry will automatically switch their portfolios*, *I may start 10% of the product with that particular logo let me say*, *then the consumer is slowly switching over to that and demanding more*, *than the ratio may change to 50–60%*, *you never know how the ratio will change over the time.” (Participant from the food industry)*

#### 5. Strengthen research related to salt (sources of salt in Indian diet, actual consumption of salt)

According to the academia, physicians, and government stakeholders, there is need to generate data on country and state-specific actual dietary salt intake and different food sources contributing to the population intake. They indicated that these data would help in the implementation and monitoring of a salt reduction strategy in India.

*“First of all we really don’t have very good data on what is the source of salt and all*. *It is an assumption that it is more due to home-made food*, *which probably may hold true for the rural area but I am not sure about the urban areas*. *So first we need to collect these data on what are the sources of salt and then plan accordingly*.*” (Participant from the government)*

Few of the participants indicated that salt substitution could be one possible technique to decrease sodium and increase other components of salt like potassium and magnesium chloride, but they reported the lack of research in this area.

*“The best way of reducing salt intake is by increasing saltiness*; *all the chloride salts have saltiness*. *Do a food safety analysis of all the chloride salts*; *you will find some are safe and some are unsafe*. *Pick up which are good*, *do the formulation of salt if add 2% of this salt that is Magnesium Chloride (MgCl) to Sodium chloride (NaCl)*, *saltiness of salt will increase what will happen is that if earlier I was taking half spoon now I am taking less salt as saltiness has increased*. *So this way it can be done*.*” (Participant from the food industry)*

**Fig 1:**
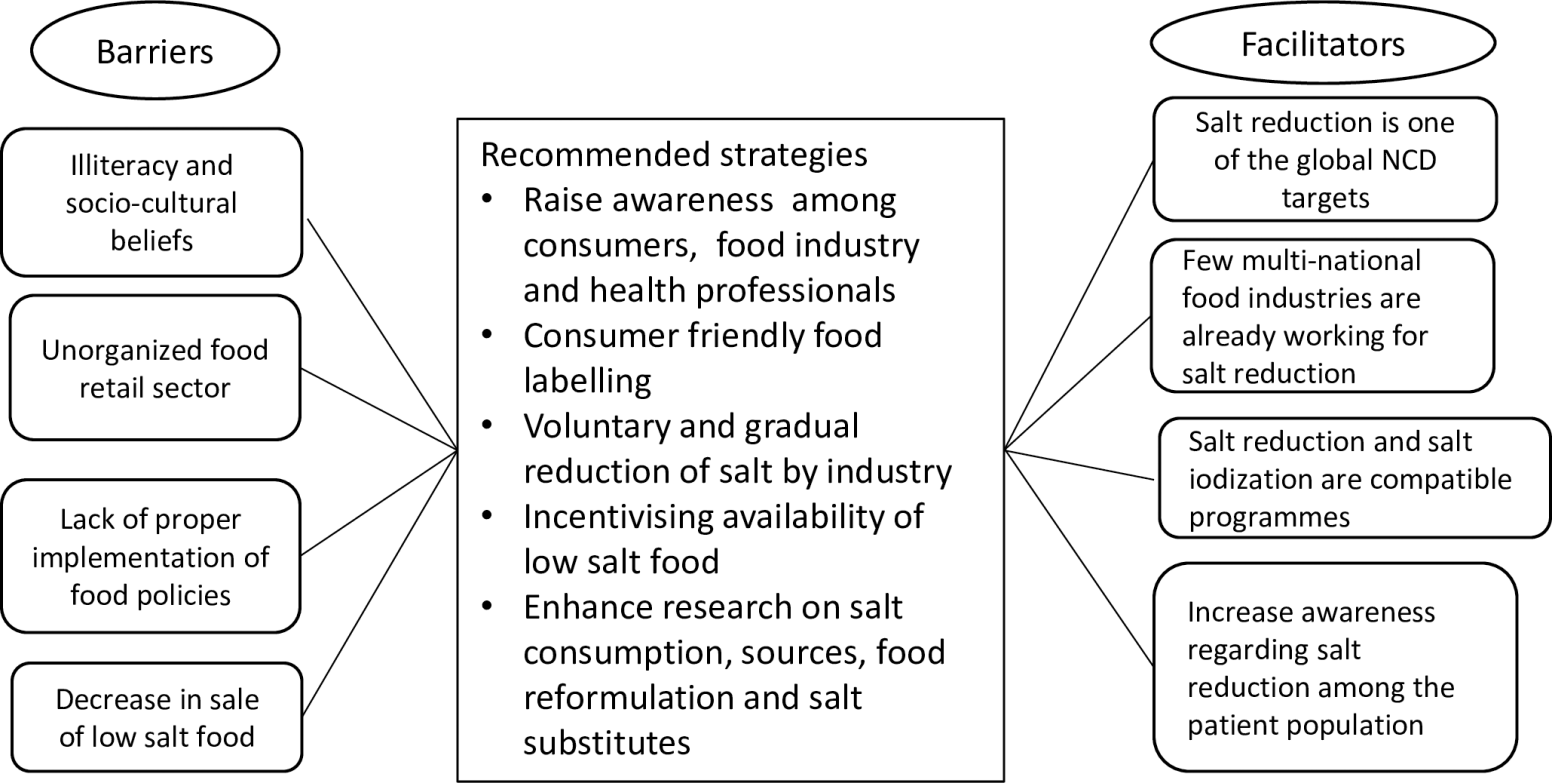
A Potential framework for initiating salt reduction in India.

Based on our findings, we have developed a framework highlighting the key strategies and issues to initiate a salt reduction effort in India, which is summarized in Fig 1.

## Discussion

To the best of our knowledge, this is the first study to examine key stakeholders’ perspectives regarding a potential salt reduction strategy in India. The findings highlight the major strategies and challenges that needs to be considered when developing a salt reduction strategy for implementation in India.

One of the key facilitators for salt reduction is the fact that it is already among the Global NCD reduction targets and India is the first country to adopt the global monitoring framework on NCDs and subsequently develop a national multi-sectoral action plan to implement it [18,19]. India needs to submit the progress reports of this monitoring framework to the WHO Regional Committee sessions in 2016, 2018, and 2021[20]. India has also adopted the ‘New Delhi Declaration on High Blood Pressure’. Therefore, the health ministry is evidently committed to prioritizing hypertension prevention to reduce its high burden[21]. While most of the stakeholders agreed that there is a need for an India specific salt reduction strategy, there are few stakeholders from the government who felt that there is still lack of evidence from India regarding salt reduction and its likely impact on improving hypertension control. They underlined the need for generating contemporary data on salt intake, its sources and related issues to guide salt reduction efforts. Thus, the current project which aims to address this gap is both pertinent and timely[12,22]. The planned national risk factor survey to track the NCD targets is an additional platform that can be used to generate such data as well as track trends[19].

Most of the stakeholders agreed that generating awareness among consumers, the food industry and health professionals, to be the likely first step toward initiation of salt reduction efforts. Envisaging the complexity of behaviour change, we have developed a behaviour change intervention package for salt reduction based on behaviour change wheel framework[23]s. This framework comprises of three layers. Central to this is a ‘behaviour system’ consisting of three essential conditions which make up the hub of the wheel: capability, opportunity, and motivation. Nine intervention functions are then organized around the hub to impact insufficiencies in these conditions. Seven policy categories are placed around these functions to potentially enable the desired interventions to happen.

This behaviour system will help us to understand the basis for the high salt consumption in the community and then design the interventions based on components of the behaviour that need to be changed. Table 2 explains, all the components of the behaviour change wheel and its relevance in context to salt reduction in India.

**Table 2:** Behaviour change wheel components and its relevance in context to salt reduction in India.

Behaviour change wheel components	Relevance to salt reduction
Sources of high salt intake behaviour	
1. Capability: Physical*	No role in behaviour change or salt reduction
2. Capability: Psychological	Lack of knowledge regarding ill effects of high salt intake, recommended salt intake, preparing low salt foods recipes, and importance of consuming low salt foods
3. Opportunity: Social	Diversity in culture and food consumption
4. Opportunity: Physical	Increased marketing of processed foods, low availability of low salt foods, lack of dietary advice by health providers
5. Motivation: Automatic process (involving emotions and impulses that arise from associative learning and/or innate dispositions)	Habit of eating high salt foods, reluctance to change, and low motivation to reduce salt
6. Motivation: Reflective process (involving evaluations and plans)	Limited awareness regarding the ill effects of high salt intake
	**Potential pathways**
Interventions	
1. Education	Provide public education to promote healthy eating
2 Persuasion	Regular reinforcement of public educational messages
3. Incentivisation	Incentivise food industry (tax subsidies, healthy logo branding etc.) to produce and market healthy foods low in salt
4. Coercion	Increase cost of unhealthy foods and decrease the cost of healthy foods (e.g.: by differential taxation, subsidies)
5. Training	• Upskill those who prepare food at homes, restaurants and other places by training in making low salt recipes • Provide technological assistance to processed food industry to reduce salt without compromising taste by decreasing salt in processed foods gradually
6. Restriction	Limit availability of high salt foods, especially to children (in school canteens/meals)
7. Environmental restructuring	Limit and regulate marketing and availability of unhealthy foods that are high in salt
8. Modelling	Utilize celebrities (sports persons, cinema actors) to promote healthy eating habits and its advantages
9. Enablement	Increase availability of healthy and low salt food
Policies	
1. Communications/marketing	Implement mass media campaign for public education
2. Guidelines	Develop and implement standard guidelines to limit use of salt, sugar and trans fat, as well as promote consumer friendly labelling
3. Fiscal	Increase taxes for unhealthy foods high in salt
4. Regulation	Regulate sale and marketing of unhealthy foods (in schools and educational institutions, work places, communities) as well as implement mandatory labelling
5. Legislation	Develop legislation to tax unhealthy foods, limit sale and marketing of unhealthy foods
6. Service provision	Increase the availability of healthy foods (in schools and educational institutions, work places, communities), provide health education in all healthcare institutions (through educators or nurses)
7. Environmental and social planning	Create enabling environment to make healthy food choices the default choices

*It means individual’s physical capacity to engage in the activity concerned

While changing consumer behavior was found to be the essential component of a potential salt reduction strategy, a need to generate awareness among food manufacturers, especially among the unorganized food retail sector, whose food marketing relies primarily on catering to local tastes, was also underlined. As part of educating consumers, it was felt that educational campaign should cover all the age groups, utilizing multiple communication channels and strategies.

A key point that emerged was that along with the ill effects of excess salt consumption, the awareness campaign should also educate people regarding likely ways in which food with less salt can be prepared, the correct amount of salt to be consumed daily and practical steps that can be undertaken to reduce individual consumption. The high level of awareness on the deleterious impact of excess salt intake among the hypertensive patients can be leveraged to improve awareness among others in their communities. It was also highlighted that salt reduction should be part of the larger health campaign on healthier diets, and not as a standalone effort. In all the countries with salt reduction strategies, consumer education is the most commonly used strategy followed by food reformulation by the industry[24].

With rapid urbanization in India, the intake of processed foods is also increasing. As most salt is hidden in processed food, it is likely that large amounts of salt are being consumed by consumers. Thus, there is an urgent need to reduce salt content in processed foods as well as improve food labeling, which should also be easy to comprehend and consumer friendly. Currently, in India the implementation of various food and dietary policies are suboptimal. For instance, as part of this large multi-component study we had conducted a systematic survey of processed foods in eleven retail shops in Delhi and Hyderabad and found that less than 50% of products were compliant with the Food Safety and Standards Authority of India (FSSAI) regulations and reported sodium content in the labels [25]. Further, despite multiple polices and significant achievements in improving population nutrition, many challenges still persist as evidenced by considerable childhood under nutrition rates, which now co-exists with rising levels of overweight and obesity [26] Few of the multinational food companies are already trying to develop and implement food reformulation strategies, considering the public health issues related to high sodium products. This is encouraging and will contribute to create an enabling environment for consumers to make healthier choices their default choices. In addition, it will also nudge the other companies to contemplate and eventually move towards reformulating their products.

Despite the recent controversies around the levels at which salt reduction should be warranted, no respondents highlighted this as a potential impediment to implement salt reducing programmes nationally[27].

Given the experiences in other developed countries where salt reduction has been achieved, it appears that collaboration with the food industry is a potential strategy that needs consideration according to many stakeholders. This is specifically relevant in the case of food reformulation, where the Government can help the industry to produce low salt food either by providing incentives in the form of tax exemptions or accrediting healthier alternatives with some healthy logo, which can help them for marketing the product and sustain sales as well as provide the impetus to ultimately move to large-scale reformulation across all food categories. Governments being a major food purchaser can also assist such effort by procuring only healthier (low salt) foods for its institutions and organizations, such that it will be an added incentive for the industry to sell healthier low salt foods[24]s. A systematic review of healthy food procurement policies in public institutions such as schools, government institutions, and hospitals had illustrated the positive impact on the availability and selection of healthy food[28].

Although our participants have highlighted the importance of voluntary salt reduction by the industry, worldwide, more countries are now shifting from solely voluntary initiatives to legislative initiatives such as instituting maximum sodium content limits in foods, taxation of high-sodium foods, mandatory Front of Pack Labelling (FoPL) schemes for high salt foods and sodium content standards for publicly procured foods and meals[24,29,30]. Most common FoPL schemes used are logos and symbols, especially traffic light labels [2,31]. Thus, in countries like India with illiteracy issues and different languages, we can likely start with a law around consumer friendly FoPL in popular processed foods with this being applied specifically to just the largest companies, so as to ensure the ease of enforcement. However, given that most of the salt intake is from non-processed foods, consumers education remains the cornerstone for salt reduction efforts [32].

Often, a major barrier for salt reduction is the compatibility issue with the already established iodine supplementation programme. Reassuringly, most stakeholders agreed that both the programmes are compatible, as both programmes adopt a multi-stakeholder approach that encompasses health promotion, prevention, treatment and rehabilitation, and involve the food and catering industry for effective implementation. Leveraging technology, we can increase the concentration of iodine in salt, which is currently based on the assumption of a 10g salt intake per person per day, as the salt intakes are gradually reduced in the population [33]. Since salt iodization is comparatively inexpensive with an annual cost of 0.02–0.05 USD per individual, even doubling the iodine content will not increase the cost substantially [34]. Thus, the synergies need to be harnessed to make both salt reduction and iodization work in tandem to improve population health in India.

### Strengths and weaknesses

The study aimed to assess the perspectives of various stakeholders’ regarding salt reduction and has provided hitherto unknown valuable insights into the characteristics of the policy-making environment, which can potentially guide and inform the development of an India specific salt reduction strategy. We have covered all major stakeholder groups and largely managed to obtain adequate representation from each of these interest groups. Standard qualitative analytical methods have been used to derive the findings, and we believe that the data obtained represent the authentic views of the stakeholders. The study has helped examine issues related to the development and implementation of salt reduction strategies for India, in both depth and detail. The findings along with the results of the quantitative characterization of population salt intake, its sources and the food supply system, has established the evidence base, which can be leveraged to implement and evaluate any future salt reduction efforts in consonance with the WHO salt reduction and hypertension reduction targets.

As with most qualitative methods of inquiry that entails an assessment of participants’ views and experiences, the possibility of recall bias is a limitation of this study as well, as is the interviewer’s skill. To minimize this, we used a standardized guide and all the IDIs and FGDs were conducted by the same team of well-trained researchers. Given that the aim was to elicit a broad spectrum view of different stakeholders, we tried to ensure the inclusion of key people in all the stakeholder’s groups of interest so as to reduce potentiality for bias and obtain valid findings.

## Conclusions

In this study of multiple key influential stakeholders in India, most of the respondents were in alignment with the need for a salt reduction programme to prevent and control hypertension and related CVD in India. The development and adoption of the National Multi-Sectoral Action Plan to reduce premature NCDs in India provides a potential platform that can be leveraged to drive, implement and monitor salt reduction efforts[19]. The other components of this research project have already generated contemporary data on actual population salt intake and its sources. Key stakeholders cited lack of these data as an impediment to initiate salt reduction efforts as mandated in the aforementioned action plan[13,22].

## Supporting information

S1 Appendix In-depth interview checklist. (PDF)

S2 Appendix Focus group discussion checklist. (PDF)
