# Supplementary material for: Stakeholders’ perceptions regarding a salt reduction strategy for India: Findings from qualitative research
Source: PLoS One. 2018 Aug 6;13(8):e0201707. doi: 10.1371/journal.pone.0201707 (PMC6078292; doi:10.1371/journal.pone.0201707)
Supplement: S1 Appendix — (PDF) [file pone.0201707.s001.pdf]

APPENDIX E

Developing a national salt reduction program for India

FOCUS GROUP/Interview INFORMATION

**State**	
**Interview Number**	
**Stakeholder Organisations/Sectors represented in focus group (i.e., industry, health, etc.)**	
**Date**	

BACKGROUND

Participants will be asked to suggest which actions need to be taken in terms of salt reduction to reduce the high burden of cardiovascular disease in India. In addition, they will be asked to comment on pre-defined interventions. Participants will be given background information about existing policy commitments nationally and regionally, and relevant evidence on interventions. This will help inform the focus group discussions.

INTRODUCTION

Thank you for taking the time to join our focus group discussion/to be interviewed. The aim of this focus group/interview is to discuss salt intakes and salt related actions/policy interventions in India. We want to get your opinions about different interventions to reduce salt consumption and how useful they might be.

We would also like to remind you that this discussion/interview is being audio-recorded.

FOCUS GROUP/Interview TOPICS - CONSUMERS

MAIN FOCUS: What can be done to change consumer behaviour?

1. ACTION

- What actions would encourage you to reduce the amount of salt you add in cooking? What actions would encourage you to reduce the amount of salty foods and snacks you buy?

- What help do you need to reduce your salt intake (through home prepared foods, processed foods and meals outside home)

2. FEASIBILITY

- What would make it difficult for you to use less salt in cooking?
- What would make it difficult to buy less of the very salty foods?
- Are there any other issues that you would like to highlight in relation to salt reduction that have not already been covered?

FOCUS GROUP/Interview TOPICS - INDUSTRY

MAIN FOCUS: What can industry do to reduce salt in their food products?

1. ACTION

- What actions need to be taken to decrease sodium levels in processed foods sold/available in India? How should it be done?
- Who should take the lead? Who should be involved?
- What should be the timescale?
- What are the barriers related to reducing salt intakes and salt levels in foods?
- What are the opportunities related to reducing salt intakes and salt levels in foods?

2. FEASIBILITY

- Would reducing salt levels in foods be feasible?
- Would reducing salt levels in foods be low cost to do?

3. ACCEPTABILITY

- Would reducing salt intakes and salt levels in foods be acceptable to your consumers do you think?
- What could be done to increase acceptability to consumers?

4. IMPACT

- Would the proposed actions/interventions be effective? Why?
- Are there side effects associated with the actions/ interventions?
- How would you prioritise the proposed actions/policy interventions? Why?

5. ADDITIONAL INFORMATION

- Are there any other issues that you would like to highlight in relation to salt reduction that have not already been covered?

FOCUS GROUP/Interview TOPICS - GOVERNMENT

MAIN FOCUS: What is government's role (i.e., trade, commerce, marketing, food controls, licensing) in reducing salt intakes?

1. ACTION

- What actions need to be taken to decrease sodium/salt intake?
- Who should take the lead? Who should be involved?
- What are the barriers (i.e., trade, commerce, etc.) related to reducing salt intakes and salt levels in foods and diets?
- How can sectors outside of health help facilitate salt reduction?
- What are the opportunities related to reducing salt intakes and salt levels in foods and diets?

2. IMPACT

- Would the proposed actions/interventions be effective? Why?
- Are there side effects associated with the actions/ interventions?
- How would you prioritise the proposed actions/policy interventions? Why?

3. ADDITIONAL INFORMATION

- Are there any other issues that you would like to highlight in relation to salt reduction that have not already been covered?
