# Supplementary material for: Stakeholders’ perceptions regarding a salt reduction strategy for India: Findings from qualitative research
Source: PLoS One. 2018 Aug 6;13(8):e0201707. doi: 10.1371/journal.pone.0201707 (PMC6078292; doi:10.1371/journal.pone.0201707)
Supplement: S2 Appendix — (PDF) [file pone.0201707.s002.pdf]

SALT PROJECT, George Institute for Global Health, India

Questionnaire for FGD (Interview) for the Consumers

INTRODUCTION

1. What is your general daily diet?
2. In your opinion, generally, how much salt is used in your cooking?
3. In your understanding, what are the major food sources that are high in salt?
   a. what are reasons for using high salt in the preparations?
   b. Why do you like these mentioned foods?
4. What does eating a lot of salt in your daily diet do?
   a. Could it lead to anything?
5. How important do you think reducing salt intake in your diet is?
   a. why so? (do you think reducing salt would have positive effect to your health?)

ACTION

6 What action would encourage you to reduce the amount of salt you add in cooking? (Awareness to the cooks or decision makers in the family? (Prob Q.while cooking/buying/eating out?)

a. What action would encourage you to reduce the amount of salty foods and snacks you buy? (Media's role, Doctors advice, traffic light labelling system?)

b. What action would encourage you to cutting habit of using salt at the table?

7 When buying packaged food items, how often do you look for the nutrition information?

a. Do you specifically look for the salt content?

b. Do you think it would be helpful to have information about the salt content on food packages? (Talk about food switch)

8 What type of programs/policies should be made by the government for salt reduction among the population?
a.Media role?

b. Food industry role?

c. Resturants/hotels/street vendors role?

(Examples such as labelling sodium content in food products, consumer awareness, development of symbols to identify low salt products, agreements with food industry to lower the salt content of food products, and regulations).

FEASIBITILY

9. What would make it difficult for you to use less salt in cooking? (Family members like the older people's preference, children's preference, and too used to certain palate level)

   a.What would make it difficult for you to cutting habit of using salt at the table?

   b. What would make it difficult to buy less salty foods ? (non-availability of low salt items, high price, preference)

   c. What would make it difficult for you to choose less salt in eating out? (Family members' preference of salt level in food, children presence to go out and eat?

Exit questions:

10. Are there any other issues/suggestions for reducing salt intake among population?
